# Survey of medication history of patients with stroke after discharge from an acute hospital ward: a case series study

**DOI:** 10.1186/s40780-025-00415-8

**Published:** 2025-02-03

**Authors:** Yuko Fukuda, Risa Ito, Misaki Kakihana, Tsutomu Takahashi, Tetsuji Kanemoto, Toshiyuki Sahara, Masahiko Tsujikawa, Mitsuko Onda

**Affiliations:** 1 Department of Pharmacy Hoshigaoka Medical Center Japan Community Healthcare Organization (JCHO), 4-8-1, Hoshigaoka, Hirakata, Osaka 573-8511 Japan; 2 Department of Social and Administrative Pharmacy, Faculty of Pharmacy, Osaka Medical and Pharmaceutical University, 4-20-1, Nasahara, Takatsuki, Osaka 569-1094 Japan; 3 Department of Stroke Medicine Hoshigaoka Medical Center Japan Community Healthcare Organization (JCHO), 4-8-1, Hoshigaoka, Hirakata, Osaka 573-8511 Japan; 4 The Hirakata City Pharmacists Association, 3F, Doctor’s Hall, 2-14-16, Kinnyahonnmachi, Hirakata, Osaka 573-1197 Japan; 5 Division of Medical Care, Department of General Affairs, JCHO West Japan Regional Office, 4-2-78, Fukushima, Fukushima-Ku, Osaka, Osaka 553-0003 Japan

**Keywords:** Stroke, Discharge, Medication history, Prescription continuity, Pharmacist collaboration, Seamless care

## Abstract

**Background:**

Stroke is a leading cause of death, reducing disability-free life expectancy. After acute treatment, patients require rehabilitation to prevent recurrence. Continued use of medication is crucial for recurrence prevention and risk management, even after the transition from acute-care institutions to other medical institutions. Although “discharge summaries on medications” are shared between hospitals and community pharmacists, no reports have addressed medication continuity for patients with stroke transferred to other institutions after discharge. This study aimed to clarify medication continuity, particularly for medications adjusted during hospitalization that should be continued even after discharge, by investigating the medication use histories of patients with stroke transferred from acute care hospitals to outpatient hospitals.

**Methods:**

We enrolled patients who were discharged from an acute ward between June 11, 2022, and March 31, 2023, after receiving inpatient care at the Japan Community Healthcare Organization, Hoshigaoka Medical Center for acute stroke, and transferred to other outpatient hospitals. This study was conducted between June 2022 and April 2023. We extracted and assessed prescription continuity and carefully examined clinically relevant discrepancies after comparing the discharge prescription with that at the first outpatient visit.

**Results:**

Of the 42 patients enrolled, seven (16.7%) had one or more discrepancies involving 13 medications. Based on the medicinal efficacy classification, four patients treated with other blood and body fluids-related agents (antiplatelet drugs), three patients treated with agents for hyperlipidemia (statins), two patients with agents for peptic ulcers, two patients with vasodilators, one patient treated with antihypertensives, and one patient with other agents affecting digestive organs (antiemetic agents that acts on the central nervous system) had discrepancies. Furthermore, discrepancies in medication discontinuation or reduction recommended by a stroke specialist, which may increase the risk of stroke recurrence, were identified in five patients (seven drugs: four antiplatelet drugs and three statins). Of 13 discrepancies, community pharmacists inquired about 3 cases with physicians, none were approved.

**Conclusion:**

The medication to prevent stroke recurrence might not be continued after transit to another outpatient after discharge. Reconsidering patient information sharing between hospital and community pharmacists and establishing a more strengthened sharing system is necessary to achieve seamless pharmacotherapy.

## Background

Cardiovascular diseases, including cerebrovascular diseases, account for the highest proportion of national healthcare expenditure in Japan [1]. Stroke, in particular, is the fourth leading cause of death and contributes to 19% of long-term care needs [2, 3].

After the onset of stroke, patients are usually provided with professional hyper-acute care and medication immediately to prevent recurrence, along with a review of pre-admission prescriptions in acute-care institutions. Depending on the patient's condition, treatment continues with transitions to a convalescent-phase or maintenance-phase medical institution (including a long-term care hospital, clinic, or patient's home). During these transitions, patients require education on general lifestyle, diet, and medication guidance, as well as recurrence risk management and appropriate rehabilitation, even after acute treatment, to prevent recurrence. Therefore, coordinated, continuous care across healthcare provider facilities is essential to ensure the provision of comprehensive medical services to patients [4].

When patients transit from acute-care institutions to other medical institutions, physicians in charge of acute-care institutions inform those in other medical institutions of the necessary patient information using medical information sheets. In addition, regarding patients with stroke, treatment details are taken over from the acute phase through the convalescent and maintenance phases among medical institutions, where patients receive treatment using “the liaison critical pathway for stroke” to ensure continuity of care. However, utilization rates in this pathway vary among medical institutions [5].

In other countries, studies have reported undesirable outcomes after hospital discharge [6, 7]. Forster et al. reported the incidence, severity, preventability, and amelioration of adverse events post-discharge, noting that adverse drug events were the most common, with poor communication between hospital caregivers and either the patient or primary care physician (59% of preventable and ameliorable adverse events) [6]. Moor et al. reported that medication continuity errors were the most common issue when patients transitioned from a hospital to an outpatient setting (primary care practice) [7]. Such studies have not been conducted in Japan, although Shinmori et al. emphasized the need to assess this problem in Japan and discuss strategies tailored to the Japanese healthcare system [8].

In recent years, sharing patient information, including medication reconciliation and reasons for prescriptions during hospitalization, through “discharge summaries about medication” has been implemented between hospital and community pharmacies. Therefore, family pharmacists are expected to provide more effective centralized pharmaceutical care management. Several self-administered pharmacist surveys have highlighted the usefulness of these summaries [9–11], citing increased efficiency in obtaining patient information, improved medication instructions, and increased prescription inquiries. However, it remains unclear whether sharing patient information using discharge summaries effectively prevents medication errors such as “the omission of medication which should be continuously administrated post-discharge.” Moreover, it is unknown whether continuity of medication for the prevention of recurrent stroke is ensured in stroke patients discharged from acute care hospitals and transferred to other hospitals as outpatients.

Therefore, this study aimed to clarify prescribing continuity for medication adjusted during hospitalization and necessary for continuation post-discharge by investigating the medication use histories of patients with stroke transferred from acute care hospitals to other outpatient hospitals.

## Methods

### Aim, design, setting, and participants

This case series study aimed to clarify prescribing continuity for medication adjusted during hospitalization and necessary for continuation post-discharge by investigating the medication use histories of patients with stroke transferred from acute care hospitals to other outpatient hospitals. Patients discharged from the acute ward of the Japan Community Healthcare Organization (JCHO), Hoshigaoka Medical, between June 11, 2022, and March 31, 2023, after in-patient care for acute stroke and transferred to other outpatient hospitals were enrolled. This study was conducted between June 2022 and April 2023.

### Inclusion and exclusion criteria

Patients diagnosed with cerebral infarction, transient ischemic attack, cerebral hemorrhage, or subarachnoid hemorrhage, aged ≥ 18 years at the time of consent, and who provided written informed consent to participate in the study were included.

Patients without family pharmacies, who were not prescribed any medication at discharge, and who were deemed unsuitable by the study investigator or collaborators were excluded.

### Survey method

At the patients’ discharge, the physician's medical information sheets were handed over to all patients, patients or patients’ families are instructed to give them to their family physicians when they visit the outpatient clinic. Hospital pharmacists made the discharge summaries on medications for the patients whose prescriptions were changed or discontinued compared to before admission and the summaries was faxed to the family pharmacies from the Community Relations Office the day after the discharge date (next business day).

A hospital pharmacist, who was one of the study investigators, contacted patients’ family pharmacies by phone following their first outpatient visit after discharge. Pharmacies were asked to fax medication information forms and other documents containing outpatient prescriptions. The date of the first outpatient visit was predicted based on the duration of the discharge prescription.

### Assessment of prescription continuity

We extracted and assessed prescription continuity and carefully examined clinically concerning discrepancies (Hereinafter referred to as discrepancies) after comparing the discharge prescription with that at the first outpatient visit after discharge. Changes in prescriptions between discharge and the first outpatient visit were classified into five groups: (1) drug discontinuation, (2) drug addition, (3) dosage changes (same drug), (4) usage changes (same drug), and (5) drug substitutions. The drugs involved in the changes were identified based on the medicinal efficacy classification (87 drugs and related commodities of the Japan Standard Commodity Classification) [12].

Discrepancies were defined as the cases classified into one of the four types shown in Table 1 and were evaluated based on the opinions of physicians specializing in stroke. The primary endpoint was the proportion of patients with identified discrepancies, while the secondary endpoint was the number of discrepancies categorized by medicinal efficacy classification.

**Table 1:** Definition of discrepancy

Type 1: Discontinuation of medication recommended in the medical information prepared by the physicians specializing in stroke management (potentially increasing the risk of stroke recurrence)
Type 2: Re-prescription of a drug discontinued because it was suspected to cause side effects during hospitalization
Type 3: Re-prescription of a drug discontinued because the risk of side effects exceeded the benefits during hospitalization
Type 4: The reason (evidence) for adding, changing, or discontinuing a drug is unknown

## Results

### Patient characteristics

156 patients were discharged from the acute care unit after being diagnosed and treated with acute stroke. The subsequent locations of these patients following discharge were as follows: death at the hospital(7patients), transferred (hospitalized) to another hospital (13 patients), outpatient at their own facility (32 patients), and outpatient at another hospital (104 patients). Of the patients treated as outpatients at other hospitals, those without a family pharmacy were excluded (49 patients). The underlying reasons for the absence of a family pharmacy for these patients included the following: the medical institution did not order out-of-hospital prescriptions (34 patients), the patients were in a geriatric health care facility where medication was administered on-site (4 patients), and the family pharmacies were unknown at the time of discharge because patients looked for them after discharge (16 patients). A patient was excluded from the study due to their unwillingness to accept treatment after hospitalization and their lack of a trusting relationship with the medical institution. Additionally, six patients were unable to provide consent due to cognitive functional problems, lack of decision-making capacity, and the absence of a substitute. A total of 48 patients met the eligibility criteria and did not fall under the exclusion criteria, but one patient left the hospital before the researcher had the chance to explain the consent process.

**Fig. 1:**
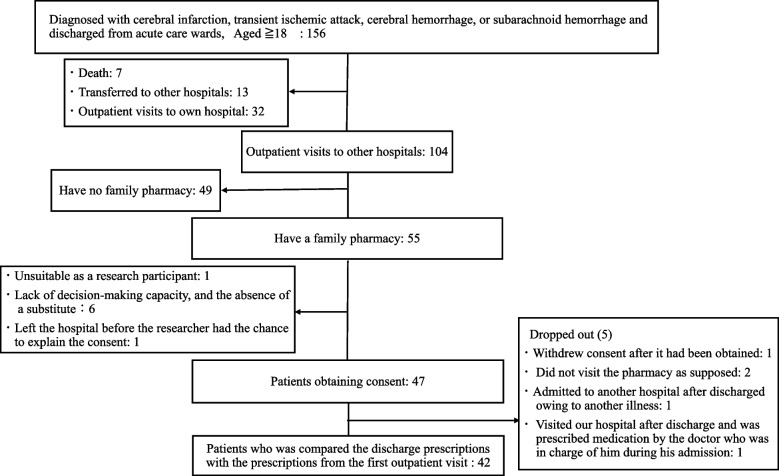
Details of patients who met the eligibility criteria and the exclusion criteria

We introduced the study to patients who met the inclusion criteria during the target period and obtained consent from 47 patients. We compared the discharge prescriptions with the prescriptions from the first outpatient visit for the 42 patients. Five patients dropped out for the following reasons: one withdrew consent after it had been obtained; two did not visit the pharmacy as supposed; one was admitted to another hospital after being discharged owing to another illness; and One was visited our hospital after discharge and was prescribed medication by the doctor who was in charge of him during his admission despite planning to visit other outpatient not our hospital prior to discharge. The reason for visiting our hospital was unclear (Fig. 1).

The background information of the 42 patients, excluding those who dropped out, is presented in Table 2. The most common diagnoses made during hospitalization were cerebral infarction (n = 31), transient ischemic attack (n = 9), and cerebral hemorrhage (n = 2). The median total score (5–35 points) for the five cognitive items of the functional independence measure, which indicates the cognitive status of patients' activities of daily living, was 30 points [13]. Regarding stroke risk factors (multiple answers possible), 35 patients had hypertension, 10 had diabetes or impaired glucose tolerance, 24 had dyslipidemia, and 15 had arrhythmia. After discharge, 38 patients returned home, and four were transferred to nursing homes or other facilities.

**Table 2:** Patient background information

Item	
Sex (n)	
Male	25
Female	17
Age (years)	
Median (IQR)	78 (73–86)
Hospitalization diagnosis (n)	
cerebral infarction	31
transient ischemic attack	9
cerebral hemorrhage	2
Cognitive FIM* score (5–35 points)	
Median (IQR)	30 (24–35)
Environment for recuperation after discharge (n)	
At home	38
Nursing homes or other facilities	4

^*^FIM Functional Independence Measure

The cognitive items consisted of five categories: communication (comprehension and expression) and social cognition (social interaction, problem-solving, and memory). Each item was evaluated on a scale of 1–7.

### The department (or specialties) of the family doctor after discharge

After discharge, 41 patients were followed by the same doctor before admission after discharge. One patient was changed family doctor after discharge. The department (or specialty) of the family doctor after discharge was internal medicine: 41 (general internal medicine: 22; cardiology: 5; gastroenterology: 4; diabetology: 3; hemodialysis: 3; hematology: 1; neurology: 1; nephrology: 1; respiratory medicine: 1) and surgical department: 1 (neurosurgery: 1).

### Identified discrepancies

The average duration of discharge prescriptions for the participants was 9 days. Among the 42 patients, seven (16.7%) had one or more discrepancies involving a total of 13 drugs.

One patient whose medication was the same as before admission was included so the medication summary was not sent for the patient. Discrepancies were detected in patients whose prescriptions changed before and after hospitalization.　One of the seven patients with discrepancies saw their family physician on the day of discharge, so, the summary was sent to the pharmacy after the visit. The summaries prepared for the other patients were sent prior to the patient's visit to the pharmacy. And, for two patients (three drugs), the community pharmacist made inquiries to the physician regarding the discrepancy based on the summary.

There were discontinuation of antiplatelet drugs and anti-hyperlipidemia medications recommended by stroke specialists in 5 of 31 cerebral infarction patients (16.1%). And, in ischemic stroke patients (cerebral infarction and transient ischemic attack), the continuity of antithrombotic drugs was 90% (36/40 patients), and that of statins was 87% (20/23 patients) Table 3.

**Table 3:** The continuities of antithrombotic drugs and anti-hyperlipidemia medications in ischemic stroke patients

Drugs	Continuities (n)
Antithrombotic drug^a^	36/40
・antiplatelet drugs (1 or 2 drugs)	24/28
・anticoagulant drugs	13/13
Anti-hyperlipidemia^b^	22/25
・HMG-CoA reductase inhibitors (statins)	20/23
・The others;	
Anti inhibitor of intestinal cholesterol transporter	5/5
Eicosapentaenonic Acid (EPA) drugs or Docosahexaenoic Acid (DHA) drugs	2/2
fibrate drugs	1/1

^a^antithrombotic drug: One patient was prescribed antiplatelet drugs and anticoagulant drugs

^b^anti-hyperlipidemia: Tree patients were taking different types of antihyperlipidemic drugs

Seven patients who had discrepancies were followed by the same family doctors before admission after discharge. And the department (or specialties) of their family doctor were general internal medicine:3, cardiology:1, hemodialysis:1, nephrology:1, neurology: 1.

Based on the medicinal efficacy classification (87 drugs and related commodities of the Japan Standard Commodity Classification), the discrepancies involved the following: four patients taking other blood and body fluids-related agents (antiplatelet drugs), three patients taking agents for hyperlipidemia (3-hydroxy-3-methylglutaryl-CoA lyase (HMG-CoA) reductase inhibitors (statins)), two patients taking agents for peptic ulcer, two patients taking vasodilators, one patient taking antihypertensives, and one patient taking other agents affecting digestive organs (antiemetic agent that acts on the central nervous system).

Table 4 shows the discrepancies identified in seven patients, categorized by the definition of discrepancy (Table 1). “Type 1” was identified in five patients (seven drugs), “Type 2” in zero patients (no extraction), “Type 3” in one patient (one drug), and “Type 4” in four patients (five drugs).

**Table 4:** Details of patients with discrepancies in medication continuity

Patient No	Diagnosis for hospitalization	Categories	Medicinal efficacy classification	Definition^a^	Details	Extra Information
1	Cerebral infarction	Addition of drugs	Other agents relating to blood and body fluids (antiplatelet drugs)	1	Aspirin was added to cilostazol	・Be prescribed single antiplatelet drug for having cerebral microbleeds
		Dosage (same drugs)	Vasodilators	4	The dose of amlodipine was increased from 2.5 mg to 5 mg	
		Dosage (same drugs)	Agents for peptic ulcer	4	The dose of lansoprazole was increased from 15 to 30 mg	・Prescribing PPI to prevent aspirin-induced peptic ulcers
2	Cerebral infarction	Drug discontinuation	Antihypertensives	4	Doxazosin 1 mg, which was added during hospitalization, was discontinued	
3	Cerebral infarction	Dosage (same drugs)	Agents for hyperlipidemia (HMG-CoA reductase inhibitors (statins))	1	The dose of pitavastatin was reduced from 2 to 1 mg pitavastatin	
4	Cerebral infarction	Drug discontinuation	Other agents relating to blood and body fluids (antiplatelet drugs)	1	Clopidogrel was not prescribed. *The community pharmacist made prescription inquiry to the physicians the cases but non approved by the physicians*	
		Drug discontinuation	Agents for hyperlipidemia (HMG-CoA reductase inhibitors (statins))	1	Rosuvastatin was not prescribed	
		Dosage (same drugs)	Agents for peptic ulcer	4	The dose of rabeprazole has been reduced from 20 to 10 mg	・Considering the symptoms that suggest a recurrence of peptic ulcer, the PPI dose was increased
5	Transient ischemic attack	Addition of drugs	Other agents affecting digestive organs (antiemetic agent that acts on the central nervous system)	3	Discontinued metoclopramide has been resumed	
6	Cerebral infarction	Dosage (same drugs)	Other agents relating to blood and body fluids (antiplatelet drugs)	1	The dose of clopidogrel has been reduced from 75 to 50 mg (the remaining drug was taken as prescribed before admission)	
		Addition of drugs	Vasodilators	4	2.5 mg of amlodipine was added	・She Visited her home doctor on the day of discharge
7	Cerebral infarction	Drug discontinuation	Other agents relating to blood and body fluids (antiplatelet drugs)	1	Aspirin was not prescribed. *The community pharmacist made prescription inquiry to the physicians the cases but non approved by the physicians*	・Taking 14 medications before admission and this number increased owing to recurrent stroke ・The interrupted Aspirin was resumed at the second follow-up visit
		Dosage (same drugs)	Agents for hyperlipidemia (HMG-CoA reductase inhibitors (statins))	1	Pravastatin has been reduced from 15 to 5 mg. *The community pharmacist made prescription inquiry to the physicians the cases but non approved by the physicians*	

*HMG-CoA* 3-hydroxy-3-methylglutaryl-CoA lyase, *PPI* Proton-pump inhibitor

^a^Definition of discrepancy:

1. Medication recommended in the medical information prepared by the physicians specializing in stroke is discontinued (potentially increasing the risk of stroke recurrence)

2. A drug discontinued because it was suspected to cause side effects during hospitalization has been re-prescribed

3. A drug discontinued because the risk of side effects exceeded the benefits during hospitalization has been re-prescribed

4. The reason (evidence) for adding, changing or discontinuing a drug is unknown

#### Discontinuation of medication recommended in the medical information prepared by the physicians specializing in stroke (potentially increasing the risk of stroke recurrence)

Patient No.1 had cerebral microbleeds and infarction. The specialist prescribed a single antiplatelet agent (cilostazol) at discharge because no stenosis or occlusion of the carotid artery or a major intracranial artery was observed; he thought strong antithrombotic therapy should be avoided owing to concerns about the risk of cerebral hemorrhage. These circumstances were included in the patient’s medical information sheet. However, for the first outpatient prescription post-discharge, aspirin was added to cilostazol.

Patient No.3 had stenosis and plaques in the left carotid artery and was diagnosed with cerebral infarction caused by an atherogenic embolus. The specialist considered lipid management necessary to prevent recurrence and prescribed pitavastatin 2 mg/day. As the low-density lipoprotein cholesterol (LDL-C) level after starting pitavastatin was well-controlled at 103 mg/dL, the specialist asked the family doctor to continue the prescription. However, in the first outpatient prescription post-discharge, the pitavastatin dose was reduced from 2 to 1 mg/day.

Patient No.4 was diagnosed with an atherothrombotic cerebral infarction caused by stenosis of the left internal carotid artery and multiple cerebral arteries. The specialist planned 1–2 months of dual antiplatelet therapy (DAPT) and lipid management with rosuvastatin (to adjust LDL-C to below 70–80 mg/dL). However, the first outpatient prescription post-discharge was a single antiplatelet drug (aspirin only); therefore, DAPT was completed earlier than the specialist-recommended treatment period. Rosuvastatin was not prescribed, although no adverse effects were observed from the statins. The pharmacist at the family pharmacy inquired with the family doctor regarding the omission of clopidogrel prescriptions based on the discharge summary; however, the doctor replied that no changes were needed, and the reasons for this were unclear.

Patient No.6 had a history of cerebral infarction and had been taking clopidogrel 50 mg/day; however, the patient still had recurrent cerebral infarction. After hospital admission, the specialist increased the clopidogrel dose to 75 mg/day, which is the usual dose. However, after discharge, her family doctor instructed her to take the remaining clopidogrel prescribed before admission; therefore, she could only take 50 mg/day of clopidogrel instead of 75 mg/day.

Patient No.7 was diagnosed with cerebral infarction with stenosis of the carotid and intracranial arteries. The patient had another stroke 3 years ago and had been taking clopidogrel 75 mg/day and pravastatin 5 mg/day. Owing to the advanced cerebral atherosclerosis compared with the initial stroke, the specialist considered that the risk of recurrent stroke was high and initiated DAPT (clopidogrel plus aspirin) while increasing the dose of pravastatin to 15 mg/day. On discharge, a medical information sheet was written to recommend a few months of DAPT, with careful monitoring for hemorrhage and maintaining the increased pravastatin dose at 15 mg/day. However, at the first outpatient prescription post-discharge, the antiplatelet medication was changed to a single agent of clopidogrel 75 mg/day, and the statin was reduced to pravastatin 5 mg/day, which was returned to the prescription before the recurrent stroke. The pharmacist at the family pharmacy inquired with the family doctor about the absence of aspirin on the prescription and the reduction in the pravastatin dose. The doctor replied that the patient might have difficulty managing the increased number of tablets, and the prescription inquiry was not approved.

#### Re-prescription of a drug discontinued because the risk of side effects exceeded the benefits during hospitalization

Patient No.5 was taking metoclopramide tablets before admission. As no gastrointestinal symptoms, such as vomiting, were observed during hospitalization, the pharmacist in charge of the ward proposed discontinuation to the doctor because of concerns about metoclopramide's potential side effects (such as extrapyramidal symptoms). Gastrointestinal symptoms were not observed after the discontinuation of metoclopramide; therefore, it was deemed unnecessary to restart treatment at discharge. After discharge, metoclopramide was re-prescribed during the first outpatient visit; however, no vomiting or other symptoms were observed.

#### The reason (evidence) for adding, changing, or discontinuing a drug is unknown

Patient No.1 was prescribed amlodipine increased from 2.5 mg/day to 5 mg/day, although no information regarding an increase in blood pressure was observed after discharge. In addition, the patient was also prescribed lansoprazole 15 mg/day (a proton pump inhibitor) to prevent aspirin-induced peptic ulcers. Following discharge, the lansoprazole dose was increased from 15 mg/day to 30 mg/day, although the patient had no symptoms suggestive of a peptic ulcer.

Patient No.2 was undergoing hemodialysis, and because his previous treatment for hypertension was insufficient to regulate blood pressure on non-dialysis days, doxazosin 1 mg/day was added. However, doxazosin was not prescribed by the outpatient hospital post-discharge.

Patient No.4 had a history of peptic ulcer and was taking rabeprazole 10 mg/day with aspirin before hospital admission. Rabeprazole 10 mg/day was continued after admission; however, the patient complained of stomach pain; therefore, the dose of rabeprazole was increased from 10 mg/day to 20 mg/day, indicating that the peptic ulcer might recur. Although the medical information sheet noted that the same dose was to be administered with the continuation of aspirin, the prescription after discharge reverted to the pre-admission dose of 10 mg/day.

Patient No.6 had a stable systolic blood pressure of 110–140 mmHg during hospitalization. On the day of discharge, the patient visited her family doctor, who added 2.5 mg/day of amlodipine.

## Discussion

This study found that in Japan, discrepancies in prescription at discharge and the first outpatient prescription post-discharge may have occurred in patients with stroke transferred to other outpatient clinics, and those necessary medications may be discontinued. In particular, discontinuation of antiplatelet drugs and anti-hyperlipidemia medications recommended by stroke specialists in 5 of 31 cerebral infarction patients (16.1%) suggests that recurrent stroke may be triggered by interruption of preventive management (Table 2, Table 4:Patient No.1,3,4,6 and 7).

Hohman et al. studied medication continuity after hospital discharge in patients with ischemic stroke [14]. In their study, prescription continuity was examined by comparing two groups: the control group (CG) consisted of patients whose primary physicians (PCPs) were given a discharge letter, including the medication list, by the neurologist in charge during hospitalization, and the intervention group (IG) in which the discharge letter written by the clinical pharmacist in addition to the doctor's discharge letter was given to the PCPs. The discharge letters written by the clinical pharmacist included the medication at preadmission and discharge, a detailed description of the antithrombotic drugs (indication, duration of anticoagulant treatment, and DAPT), and the reason for adding statins. Consequently, the continuity of the entire medication regime quantified as the mean of the continuity of each drug improved in the IG: (CG: 83.3% and IG: 90.9%). The continuity status by drug efficacy was as follows: for antithrombotic drugs, CG: 83.8%, IG: 91.9%; and for statins, CG: 69.8%, IG: 87.7%, with improvements particularly observed in the continuity status of statins.

In contrast, our study, discharge medication summaries were provided to community pharmacists by hospital pharmacists, along with collaboration among physicians at patient discharge. In the physicians' medical information, patient information, such as diagnosis, treatment progress, reasons for prescription changes, duration of DAPT, and rationale for prescribing statins (LDL-C targets), in addition to the discharge medication list, were detailed. When evaluated as a percentage of the number of patients, our result was worse than those of the CG (only medical information sheets written by physicians) reported by Hohman et al. However, when we examined the continuity of antithrombotic drugs and statins only among the participants who had suffered an ischemic stroke (cerebral infarction and transient ischemic attack), the continuity of antithrombotic drugs was 90% (36/40 patients), and that of statins was 87% (20/23 patients) (Table 3). Thus, our results are similar to those of the IG group reported by Hohman et al.

Of the 13 cases of prescription discrepancies in this study, community pharmacists made prescription inquiries to the physicians on three cases (patients No. 4 and 7); however, none of them were approved by the physicians (Table 4, Patients No. 4 and 7, the details). We believe that increasing the frequency of prescription inquiries from pharmacists to physicians, such as those made in this study, by sharing patient information between hospital and community pharmacies, along with securing more approval from physicians, can further improve prescription continuity.

Patient No. 7 was taking 14 medications before admission: however, this number increased owing to recurrent stroke (Table 4 Patient No.7, Extra Information). His family physician did not prescribe any additional medications during his hospital stay, expressing concern that an increased number of pills would reduce medication adherence. It is true, however, that poly-pharmacy may lead to an increased frequency of adverse events due to drug interactions, decreased medication adherence, and medication errors; however, poly-pharmacy also has concerns about the risk of omitting (under-prescribing)　medications that should be continued [15]. Therefore, as stated in the ‘Guidelines for the Appropriate Use of Medicines in the Elderly,’ it is necessary to consider the priority of drugs rather than simply reducing the number of drug prescriptions [16]. Although the prescription inquiries for the case were not approved at the first outpatient visit, the interrupted antiplatelet drug was resumed at the second follow-up visit (Table 4 Patient No.7, Extra Information). The reason for the resumption of prescriptions is not clear, as it was not confirmed with the family doctor directly, but it is possible that the pharmacist's inquiry may have prompted the family doctor to review the prescription content by reconfirming the medical information sheet from the specialist, ultimately leading to the re-initiation of the medication.

Active prescription interventions by community pharmacists are required to improve prescription continuity. To achieve this, reevaluation of the system that shares patient information between hospitals and pharmacies is necessary.

Fukuda et al. conducted a self-recorded survey regarding the utilization of discharge medication summaries in community pharmacies; one reason cited for their under-utilization was timeliness (they were received after the patient had visited the pharmacy) [11]. Patient No. 6 had an outpatient visit on the day of discharge, and most likely, the pharmacy had not received the discharge medication summary (Table 4 Patient No.6, Extra Information). Consequently, even if the community pharmacist can ascertain the details of the prescription at the time of discharge from the patient's medication handbook, it is challenging to determine the blood pressure trend before discharge, so making a prescription inquiry would be difficult even if a prescription discrepancy is noticed. To address this issue, patient information must be shared by the time the patient receives an outpatient visit after discharge.

To enhance patient information sharing, hospital pharmacists should provide community pharmacists with detailed information on individual patients' stroke pathology, risk factors for recurrence, and risk management methods (including target LDL-C management values based on practice guidelines) [17, 18]. Communicating the detailed pathology may enable well-founded prescription inquiries regarding antithrombotic therapy for patients with stroke accompanied by micro-bleeds, such as patient No. 1 (Table 4 Patient No.1, Extra Information). Furthermore, our study identified two cases of discrepancies in the prescription of proton pump inhibitors (PPIs) for peptic ulcer prophylaxis while aspirin was administered (Table 4 Patient No.1and 4,Extra Information). PPIs are considered effective for prophylaxis against peptic ulcers in patients using continuous non-steroidal anti-inflammatory drugs including low-dose aspirin [19]. Careful information sharing is needed regarding the management of adjunctive medications to ensure the safe continuation of recurrent stroke prophylaxis agents.

In a survey conducted by Fukuda et al., community pharmacists expressed a desire to understand the type of information hospital pharmacists needed [11]. If hospital pharmacists can tell community pharmacists what kind of follow-ups and feedback they want after discharge, bidirectional communication between hospitals and pharmacies can be achieved. In addition, if hospital and community pharmacists can work together to assess patients' post-discharge prescriptions, this would allow pharmacists to intervene in cases where difficult to do without a detailed understanding of the patient's condition, such as in patients No. 4 and 1. This would probably increase the likelihood of physicians agreeing with the pharmacists' recommendations.

As shown in Fig. 1, We founded that many patients (Outpatient visits to other hospitals: 49/104) have no family pharmacy. Prescribing continuity in these patients could not be ascertained, but it would be expected that similar prescribing non-continuity would exist. Approximately 70% (34/49) of the patients who transitioned to outpatient care at other hospitals who were excluded in this study had a medical institution that did not order out-of-hospital prescriptions as their family physician. Pharmacist intervention for these patients may be difficult at present. However, for patients who did not have a family pharmacy prior to admission, it might become possible to provide patient information if the hospital pharmacist could be informed their family pharmacy from them or their family pharmacists.

In the future, we would like to establish a bidirectional relationship between hospitals and community pharmacists to address these issues and examine how this affects the continuity of prescriptions for patients with stroke before and after discharge.

### Limitation

This study had some limitations. First, the number of patients with hemorrhagic stroke (cerebral and subarachnoid hemorrhages) was small; therefore, we were unable to evaluate the continuation of prescriptions for these patients. Second, regarding the increase and discontinuation of drugs used for treating high blood pressure in patients No. 1 and No. 2, we were unable to accurately assess their blood pressure trends post-discharge. Based on the patient's subjective information indicating no change in physical condition post-discharge, they were classified as type 4 discrepancy.

Despite these limitations, we believe that this study is an important first attempt in Japan to evaluate the continuity of prescriptions for patients with stroke transferred from acute care hospitals to outpatients at other hospitals.

## Conclusions

Our study investigated the continuation of prescriptions for patients with stroke discharged from acute care hospitals and transferred to outpatient care at other hospitals and revealed that in Japan, the medication necessary for the prevention of stroke recurrence may not be continued owing to changes in the environment for medical care.

The results of our survey raise concerns that patients transferred to outpatient care at other hospitals post-discharge may have undesirable outcomes such as stroke recurrence. Establishing a new system to share patient information between hospitals and community pharmacists is a critical area for future studies.

## Data Availability

The datasets generated and/or analyzed during the current study are not publicly available due to privacy and ethical restrictions but are available from the corresponding author upon reasonable request.

## Abbreviations

DAPT: Dual Anti-Platelet Therapy
JCHO: Japan Community Healthcare Organization
LDL-C: Low-density lipoprotein cholesterol
PPI: Proton-pump inhibitor
